# Clinical and endocrine data for goserelin plus anastrozole as second-line endocrine therapy for premenopausal advanced breast cancer

**DOI:** 10.1038/sj.bjc.6601557

**Published:** 2004-02-03

**Authors:** D P Forward, K L Cheung, L Jackson, J F R Robertson

**Affiliations:** 1 Professorial Unit of Surgery, City Hospital, Hucknall Road, Nottingham NG5 1PB, UK

**Keywords:** goserelin, tamoxifen, anastrozole, breast cancer, oestradiol, endocrine therapy

## Abstract

A total of 16 premenopausal women with metastatic breast cancer (*N*=13) or locally advanced primary breast cancer (*N*=3) were treated with a combination of a gonadotropin-releasing hormone agonist goserelin, and a selective aromatase inhibitor anastrozole. All had previously been treated with goserelin and tamoxifen. In all, 12 patients (75%) achieved objective response or durable stable disease at 6 months, with a median duration of remission of 17+ months (range 6–47 months). Four patients still have clinical benefit. Introduction of goserelin and tamoxifen resulted in an 89% reduction in mean oestradiol levels (pretreatment *vs* 6 months=224 *vs* 24 pmol l^−1^) (*P*<0.0001). Substitution of tamoxifen by anastrozole on progression resulted in a further 76% fall (to 6 pmol l^−1^ at 3 months) (*P*<0.0001). Treatment with goserelin and tamoxifen led to a 90% fall in the mean follicle-stimulating hormone (*P*<0.001). This was reversed once therapy was changed to goserelin and anastrozole. A similar initial reduction was seen in the mean luteinising hormone levels, but substitution of tamoxifen by anastrozole on progression resulted in no significant change. Goserelin and tamoxifen did not lead to any significant change in testosterone and androstenedione levels. The combined use of goserelin and anastrozole as second-line endocrine therapy produces a significant clinical response of worthwhile duration, with demonstrable endocrine changes, in premenopausal women with advanced breast cancer, and offers them another therapeutic option. Further studies involving more patients and longer follow-up are indicated.

The combined use of a gonadotropin-releasing hormone (GnRH) agonist (e.g. goserelin (Zoladex, AstraZeneca)) and tamoxifen in premenopausal women with breast cancer is an established therapeutic option – either using both agents together as initial therapy or by adding tamoxifen following initial goserelin therapy (Nicholson *et al*, 1985; Williams *et al*, 1986; Robertson *et al*, 1989a; Jonat *et al*, 1995). Goserelin alone has been shown to produce castrate levels of oestradiol (E2) (Williams *et al*, 1986) and response rates similar to oophorectomy in premenopausal women both in Phase II studies (Blamey *et al*, 1992) and in a randomised study (Taylor *et al*, 1998). An early nonrandomised clinical study suggested possible extension of the duration of response upon addition of the antioestrogen tamoxifen to goserelin in premenopausal patients with advanced breast cancer (Dixon *et al*, 1991). More recently, a meta-analysis of four studies has revealed that using the combination of goserelin and tamoxifen as initial therapy produced a significantly longer time to first progression than using goserelin alone (Klijn *et al*, 2001). The rationale for this therapy is that, having effectively rendered the patient postmenopausal with the use of goserelin, the effect of peripheral E2 production in promoting hormone-sensitive breast cancer growth is inhibited by tamoxifen, as in postmenopausal women. Indeed, the combination of goserelin and tamoxifen has been shown to produce a significantly lower concentration of follicle-stimulating hormone (FSH) than goserelin alone, and a resultant (nonsignificant) reduction in E2 (Robertson *et al*, 1989a).

Selective aromatase inhibitors (e.g. anastrozole (Arimidex, AstraZeneca)) have now become the standard second-line endocrine therapy, after the failure of tamoxifen in postmenopausal women with advanced breast cancer (Buzdar *et al*, 1998). They now challenge tamoxifen as first-line endocrine therapy for hormone-sensitive advanced breast cancer (Bonneterre *et al*, 2000; Nabholtz *et al*, 2000). Therefore, following the same logic that has led to the combined use of goserelin and tamoxifen, we now report clinical and endocrine data supporting the use of goserelin combined with anastrozole for premenopausal women with advanced breast cancer, who have progressed following treatment with goserelin and tamoxifen.

## PATIENTS AND METHODS

All premenopausal women who had been treated with goserelin and anastrozole for advanced breast cancer in the Nottingham Breast Unit were included in the study. A total of 16 such patients with a mean age of 44 years (range 32–52 years) at the time of commencing goserelin and anastrozole were identified in the period 1997–2000. All of them had histologically proven breast cancer, and were treated in a dedicated Advanced Breast Cancer Clinic. They had all previously been treated with goserelin and tamoxifen, and had had a clinical benefit (see below). The median duration of partial response (PR) on goserelin and tamoxifen was 52.5 months (range 27–59 months), and that of durable stable disease (SD) was 23 months (range 10–84 months). The indications for treatment were metastatic disease (*n*=13) or locally advanced primary breast cancer. In all, 14 patients had oestrogen receptor (ER)-positive tumours. One had ER-negative tumour and one unknown ER status. Progesterone receptor status was not routinely done in this unit.

The treatment was changed to goserelin and anastrozole at the time of disease progression. The sites of disease when goserelin and anastrozole were commenced are summarised in Table 1

**Table 1:** Sites of disease for patients receiving goserelin and anastrozole

	***N***
Locally advanced primary disease	3
Metastatic	13
Soft tissue	1
Bone±soft tissue	6
Pleura	1
Bone+Pleura	2
Visceral	3
Total	16

.

### Follow-up and assessment of therapeutic response

Patients were followed up at 6 weeks, 12 weeks and thereafter at 3-monthly intervals. Clinical, radiological and biochemical (using blood tumour markers - CA15.3, CEA and ESR (Robertson *et al*, 1991)) assessments were performed. Clinical and radiological assessment of therapeutic response was carried out using criteria laid down by the International Union Against Cancer (UICC) (Hayward *et al*, 1977) while adhering to the British Breast Group recommendations that the minimum duration of remission should be 6 months (British Breast Group 1974). Objective response (OR) was defined as either complete response (CR) or PR. Clinical benefit was defined as OR or SD at 6 months (Howell *et al*, 1988; Robertson *et al*, 1989b; Robertson *et al*, 1997).

The median duration of clinical benefit was 15 months (range: 6–32 months). The median time to progression was 10 months (range: 2–16 months). Time to treatment failure was identical as no patients discontinued therapy for any other reasons.

### Therapy

All patients received goserelin 3.6 mg. by subcutaneous injection every 4 weeks along with anastrozole 1 mg daily. Therapy was continued until there was definite evidence of progressive disease (PD) according to UICC criteria.

The duration of response was calculated from the time of commencement of goserelin and anastrozole. Disease progression was taken as the end point for this study.

### Tumour markers

All patients had serum CA15.3 and CEA measured at routine clinic visits to aid clinical management. Remaining serum was stored and provided samples for retrospective hormone assays. Two patients with unassessable disease (due to sclerotic bony metastases) according to UICC criteria were assessed using tumour marker measurements only (Cheung *et al*, 2001).

### Hormone assays

Hormone assays were not performed routinely and have been performed retrospectively on stored serum for this study. The serum samples were stored at −20°C. Samples were subsequently retrieved and thawed. Standard assays for E2, FSH, luteinising hormone (LH), testosterone, dehydroisoandrosterone (DHES) and androstenedione were carried out in the respective Departments of Clinical Chemistry at Nottingham City Hospital (E2) and the Royal Marsden Hospital (LH, FSH, testosterone, DHES and androstenedione). The E2 assay had a detection limit of 5 pmol l^−1^ and an intra-assay precision of 10% coefficient of variation at 37 pmol l^−1^. Levels of the above hormones were measured during treatment first with goserelin and tamoxifen and subsequently with goserelin and anastrozole at pre-treatment, 3, 6 and 12 months and at subsequent visits. A total of 13 patients had a complete set of sequential serum samples available for these assays.

### Statistical method

**Figure 1:**
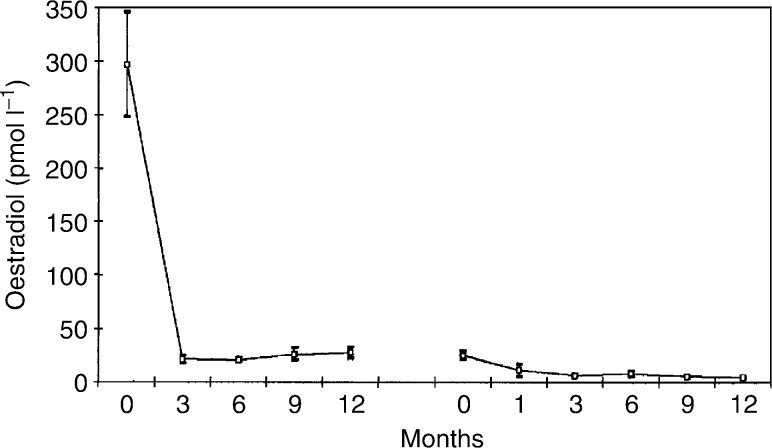
Mean (standard error of the mean) serum oestradiol levels in 13 patients treated with goserelin plus tamoxifen, followed by goserelin plus anastrozole.

Analyses were carried out using the standardised biomedical computer programme SPSS for Windows (SPSS UK Ltd). The *t*-test was used to compare pretreatment values with levels at 6 months – the standard point taken for outcome. The data are displayed graphically in Figures 1

, 2

**Figure 2:**
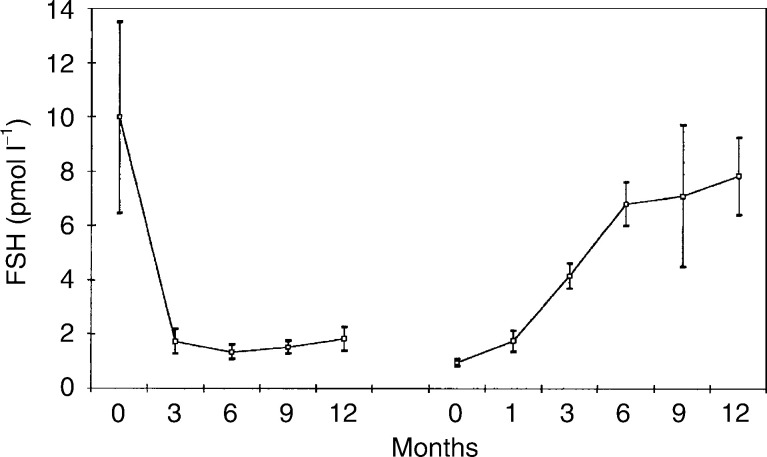
Mean (standard error of the mean) serum FSH levels in 13 patients treated with goserelin plus tamoxifen, followed by goserelin plus anastrozole.

, 3

**Figure 3:**
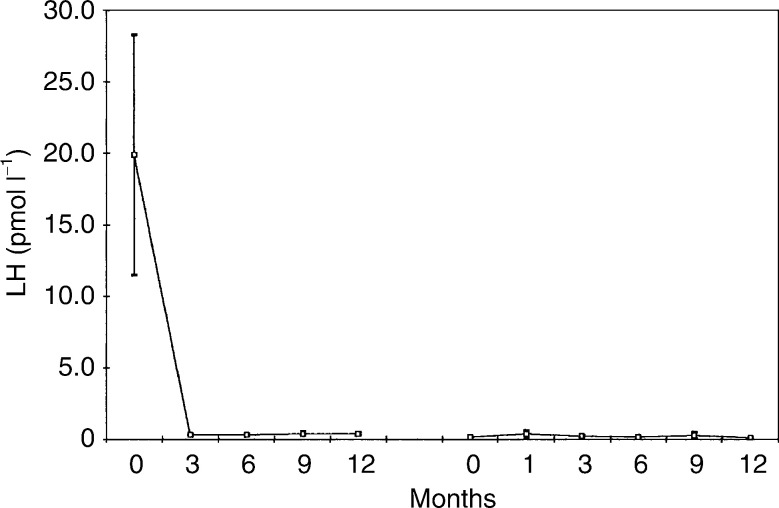
Mean (standard error of the mean) serum LH levels in 13 patients treated with goserelin plus tamoxifen, followed by goserelin plus anastrozole.

, 4

**Figure 4:**
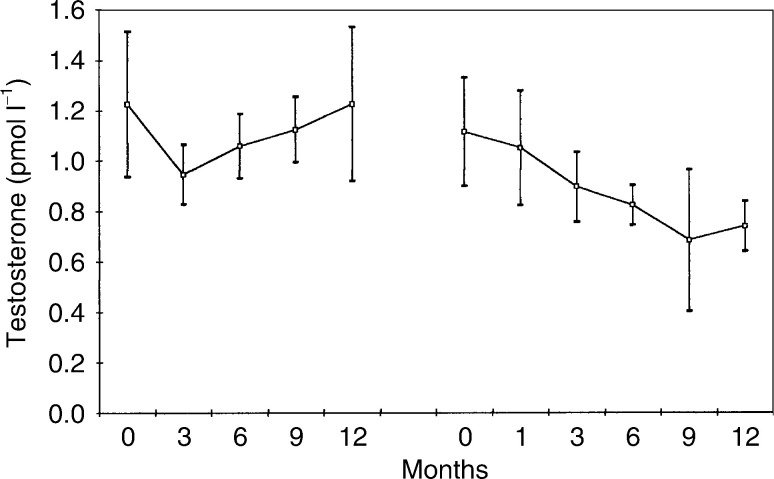
Mean (standard error of the mean) serum testosterone levels in 13 patients treated with goserelin plus tamoxifen, followed by goserelin plus anastrozole.

, 5

**Figure 5:**
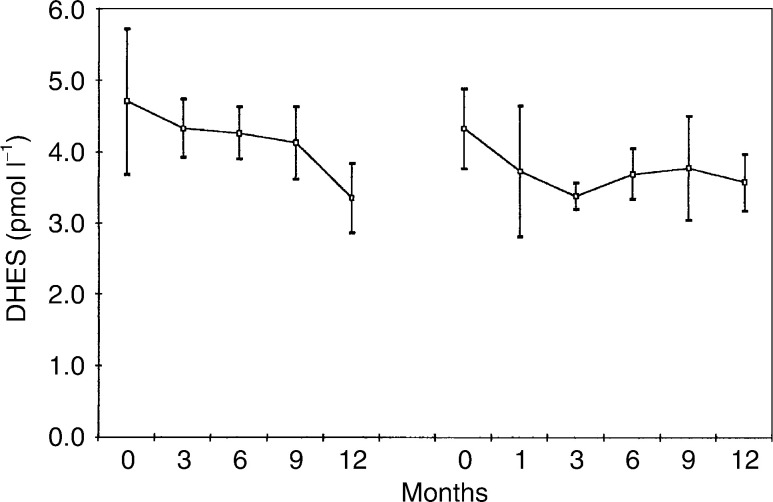
Mean (standard error of the mean) serum DHES levels in 13 patients treated with goserelin plus tamoxifen, followed by goserelin plus anastrozole.

and 6Figures 1

**Figure 6:**
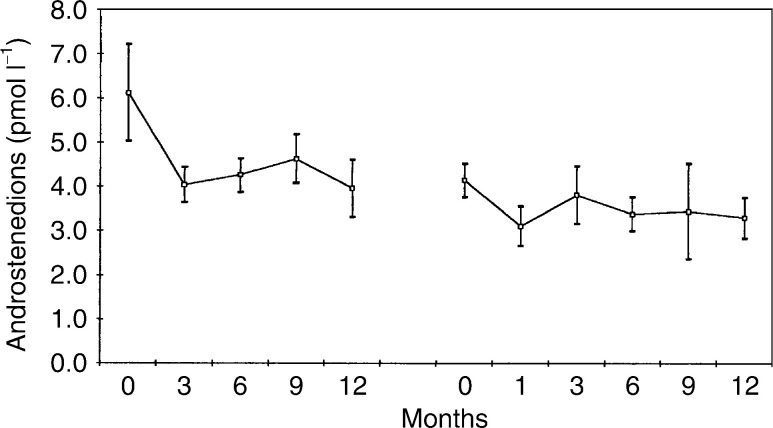
Mean (standard error of the mean) serum androstenedione levels in 13 patients treated with goserelin plus tamoxifen, followed by goserelin plus anastrozole.

. The graph represents mean values and the bars are standard errors of the mean. Statistically significant difference was defined by *P*<0.05.

## RESULTS

### Clinical data

Details of the response data are tabulated (Table 2

**Table 2:** Response and duration of therapy on goserelin and anastrozole

**UICC response at 6 months**	**No. of patients**	**Duration of therapy (months)**
PR	1	31*
SD	9	6, 11, 12, 16, 17, 17, 19*, 31, 47*
PD	4	1, 2, 3, 4
U/A	2	15*, 21

* Patients who have not yet progressed on goserelin and anastrozole. U/A, unassessable by UICC criteria.

). In all, 12 patients showed clinical benefit. These included one PR and nine SDs. A further two patients remained on goserelin and anastrozole, with no evidence of PD beyond 6 months with decreasing blood tumour markers (i.e. having a biochemical response), although the disease was unassessable by UICC criteria. The clinical benefit rate (OR+SD+biochemical response) at 6 months was therefore 75%.

Four patients progressed before 6 months (median=2.5 months). Two had pre-existing liver metastases; the third had local and pleural disease and the fourth patient had a locally advanced primary tumour.

For the 12 patients receiving goserelin and anastrozole for at least 6 months, the median duration of response was 17 months (range 6–47 months). Among these 12 patients, eight have progressed, while four continue to have clinical benefit.

The treatment was well tolerated, with no significant symptoms reported to either the clinician or the specialist breast care nurse assessing the patients. No patient discontinued therapy because of side effects of goserelin and anastrozole.

### Endocrine data

Figures 1, 2, 3, 4, 5 and 6 show the mean serum hormone levels from 13 patients for whom complete sets of sequential serum samples were obtainable. Changes in the first 12 months on goserelin and tamoxifen are shown, followed by those in the first 12 months on goserelin and anastrozole after progression.

Introduction of goserelin and tamoxifen led to an 89% reduction in E2 levels compared to pretreatment – mean concentration pretreatment *vs* 6 months being 224 and 24 pmol l^−1^, respectively (*P*<0.0001). Substitution of tamoxifen by anastrozole on progression resulted in a further 76% fall in serum E2 levels – mean concentration at pretreatment, 3 and 6 months being 24, 6 and 5 pmol l^−1^, respectively (*P*<0.0001). These figures were in agreement with laboratory reference intervals at our hospital: premenopausal levels >200 pmol l^−1^ and postmenopausal levels <110 pmol l^−1^.

Serum FSH levels also showed a significant change (Figure 2). The mean pretreatment level was 10.1 pmol l^−1^. Introduction of goserelin and tamoxifen led to a 90% fall to 1.0 pmol l^−1^ at 6 months (*P*<0.001). Once the therapy was changed to goserelin and anastrozole, there was a significant rise in the mean FSH level to 7.8 pmol l^−1^ at 12 months (*P*<0.0001).

Introduction of goserelin and tamoxifen resulted in an 89% reduction in mean LH levels (pretreatment *vs* 6 months=19.9 *vs* 0.3 pmol l^−1^) (*P*=0.01) (Figure 3). Substitution of tamoxifen by anastrozole on progression produced no significant change in LH levels.

Treatment with goserelin and tamoxifen resulted in no overall change in testosterone levels (Figure 4). Substitution of tamoxifen by anastrozole on progression led to a significant fall in mean testosterone levels from 1.06 to 0.85 pmol l^−1^ (*P*<0.05). There was a clear falling trend with goserelin and anastrozole, which had not been present during treatment with goserelin and tamoxifen.

There was no significant change in DHES levels throughout the two treatments (Figure 5).

Introduction of goserelin and tamoxifen resulted in a 31% reduction in mean androstenedione levels (pretreatment versus 6 months=6.1 *vs* 4.2 pmol l^−1^), but the fall was statistically insignificant. Substitution of tamoxifen by anastrozole on progression produced a further 18% fall from 4.2 to 3.4 pmol l^−1^ (*P*<0.02).

## DISCUSSION

In postmenopausal women with oestrogen-dependent breast carcinoma, oestrogen is suppressed both at the receptor level and by reducing circulating levels. Agents are used sequentially, for example, Tamoxifen followed by aromatase inhibitors such as anastrozole. Sequential hormone treatments for premenopausal patients with advanced breast cancer are less established. Ovarian ablation (e.g. oophorectomy or irradiation) has been used for over 100 years since Beatson first reported response to surgical oophorectomy in a patient with advanced breast cancer (Beatson 1896). Much more recently, ovarian suppression with GnRH agonist (e.g. goserelin) has been reported to produce response rates similar to ovarian ablation both in phase II studies (Blamey *et al*, 1992) and in a multicentre randomised trial (Taylor *et al*, 1998). Furthermore treatment with goserelin has been shown to result in castrate levels of E2 (Nicholson *et al*, 1985).

Tamoxifen is a well-established first-line endocrine therapy in postmenopausal women with advanced breast cancer. Megestrol acetate was the standard second-line endocrine agent after failing tamoxifen, and has now been replaced by selective aromatase inhibitors (e.g. anastrozole) (Buzdar *et al*, 1998). Recent data have shown at least equivalent effects between tamoxifen and anastrozole as first-line endocrine therapy (Bonneterre *et al*, 2000; Nabholtz *et al*, 2000). The time to progression was found to be significantly longer in patients treated with anastrozole than with tamoxifen in some of these randomised trials (Nabholtz *et al*, 2000; Milla-Santos *et al*, 2003). Similar results were also seen with other third-generation aromatase inhibitors such as letrozole (Mouridsen *et al*, 2001).

The induction of menopause using goserelin and its combination with tamoxifen is a rational extension for treating premenopausal women with advanced breast cancer (Klijn *et al*, 2001). It has been demonstrated to produce a significant OR rate of worthwhile duration. Further extension of treatment regimes in premenopausal women has led to the substitution of tamoxifen by a selective aromatase inhibitor (e.g. anastrozole) on progression of disease, just as in postmenopausal women.

### Clinical response

The results reported here show that further significant remission of worthwhile duration can be achieved using anastrozole in combination with goserelin, after tamoxifen has ceased to be effective. Clinical benefit was achieved in 12 out of 16 patients (75%) at 6 months, with a median duration of therapy of 17+ months. One of the four patients who progressed before 6 months was in fact offered chemotherapy as the treatment of choice for her liver metastases, but she refused and requested to be put on endocrine therapy. It would therefore appear that the response rate might be higher if only patients who had endocrine therapy as the appropriate treatment option were included. Although the number of patients in this series is small, the response rate has far exceeded that of traditional second-line endocrine therapy for advanced breast cancer in postmenopausal women (our previous study has shown an OR+SD rate of 53% using megestrol acetate, with a median duration of response of 15 months (Cheung *et al*, 1997)). The result in the present study represents a significant extension of disease control after progression on prior goserelin and tamoxifen, and compares favourably to the use of anastrozole in postmenopausal women (Wiseman and Adkins, 1998).

Patients in this study all had previously responded to goserelin and tamoxifen with a clinical benefit. However, it should be noted that the type of response is lower than that achieved with goserelin and tamoxifen. In the 16 patients studied, treatment with goserelin and tamoxifen resulted in one CR, five PRs and 10 SDs at 6 months. At progression, when tamoxifen was substituted by anastrozole, only one patient achieved a PR, with nine having SD. There were two patients with non-PD, as assessed by tumour marker response. There were no patients who achieved a CR. This finding is expected, as it is well known that response rates fall with each sequential endocrine manoeuvre, although the durable SD rate remains high. It must be noted that, despite a lower CR/PR rate, the clinical benefit rate (CR/PR/SD) was high, and it has been established that patients who have achieved SD for 6 months on an endocrine therapy have survival equivalent to those with CR/PR (Howell *et al*, 1988; Robertson *et al*, 1997, 1989b). A similar effect was also seen in the duration of response, which again became shortened with second-line endocrine therapy using goserelin and anastrozole.

### Endocrine response

The results for the different hormones assayed will be considered in turn. Oestradiol levels shown in Figure 1 confirm that castrate levels can be achieved with the introduction of goserelin, as previously shown (Robertson *et al*, 1989a). There was, however, a further 76% fall (*P*<0.0001) in E2 levels when tamoxifen was substituted by anastrozole (from 23.6 to 6.96 pmol l^−1^). This was associated with a good clinical response, as shown above. There were no peaks of E2 activity noted in any of the serum samples.

Follicle-stimulating hormone levels were initially suppressed by treatment with goserelin and tamoxifen. Again, this is consistent with results previously seen (Robertson *et al*, 1989a). However, substitution of tamoxifen by anastrozole led to a partial loss of this suppression, with FSH levels rising towards pretreatment values. This may be due to the effect of a negative feedback as a result of further reduction in E2 levels upon the introduction of an aromatase inhibitor. It may also be a rebound phenomenon from coming off tamoxifen.

Luteinising hormone levels were suppressed, as would be expected by constant administration of a GnRH analogue. There was no significant change in LH levels on goserelin plus anastrozole.

Testosterone levels were unchanged by treatment with goserelin and tamoxifen. Substitution of tamoxifen by anastrozole produced a 20% fall in testosterone levels (*P*<0.05). Androstenedione levels were also unchanged by treatment with goserelin and tamoxifen. Again, as with testosterone, there was a significant but small fall in hormone levels when tamoxifen was substituted by anastrozole – 18% (*P*<0.02). The levels of DHES were unaltered by treatment with either combination. It would appear that these substrates for aromatase (i.e. the precursors from which E2 is converted) do not increase with a blockade of the conversion system by the third-generation aromatase inhibitors (e.g. anastrozole).

This study has a relatively small number of patients and short overall follow-up to date. Nevertheless, it is the first study reporting on the clinical and endocrine effects of the combined use of goserelin and anastrozole as a second-line endocrine therapy for premenopausal women with advanced breast cancer. The endocrine changes are more interesting by having similar data from the same patients, while they received goserelin and tamoxifen as first-line endocrine therapy. The combination of goserelin and anastrozole produces a significant clinical benefit rate, which is also of worthwhile duration in this group of patients. Such combination therefore offers a clinically valuable therapeutic option. Further studies involving a larger number of patients and longer duration of follow-up are indicated. Such evaluation now appears to be of paramount importance, as the selective aromatase inhibitors (eg anastrozole) have recently been shown to be the preferred first-line endocrine therapy to tamoxifen in postmenopausal women with advanced breast cancer.

## References

[bib1] Beatson GT (1896) On the treatment of inoperable cases of carcinoma of the mamma: suggestion for a new method of treatment, with illustrative cases. Lancet 2: 104–107 PMC5518378 29584099

[bib2] Blamey RW, Jonat W, Kaufmann M, Bianco AR, Namer M (1992) Goserelin depot in the treatment of premenopausal advanced breast cancer. Eur J Cancer 28A (4–5): 810–814 1388037 10.1016/0959-8049(92)90120-q

[bib3] Bonneterre J, Thürlimann D, Robertson JFR, Krzakowski M, Mauriac L, Koralewski P, Vergote I, Webster A, Steinberg M, von Euler M for the Arimidex Study Group (2000) Anastrozole versus tamoxifen as first-line therapy for advanced breast cancer in 668 postmenopausal women: results of the Tamoxifen or Arimidex Randomized Group Efficacy and Tolerability Study. J Clin Oncol 18: 3748–3757 11078487 10.1200/JCO.2000.18.22.3748

[bib4] British Breast Group (1974) Assessment of response to treatment in advanced breast cancer. Lancet 2: 38 4134716

[bib5] Buzdar A, Jonat W, Howell A, Jones SE, Blomqvist CP, Vogel CL, Eiermann W, Wolter JM, Steinberg M, Webster A, Lee D (1998) Anastrozole versus megestrol acetate in the treatment of postmenopausal women with advanced breast carcinoma: results of a survival update based on a combined analysis of data from two mature phase III trials. Cancer 83: 1142–1152 9740079

[bib6] Cheung KL, Evans AJ, Robertson JFR (2001) The use of blood tumour markers in the monitoring of metastatic breast cancer unassessable for response to systemic therapy. Breast Cancer Res Treat 67: 273–278 11561773 10.1023/a:1017909727019

[bib7] Cheung KL, Willsher PC, Pinder SE, Ellis IO, Elston CW, Nicholson RI, Blamey RW, Robertson JFR (1997) Predictors of response to second-line endocrine therapy for breast cancer. Breast Cancer Res Treat 45: 219–224 9386865 10.1023/a:1005828731462

[bib8] Dixon AR, Jackson L, Robertson JFR, Nicholson RI, Blamey RW (1991) Combined goserelin and tamoxifen in premenopausal advanced breast cancer: duration of response and survival. Eur J Cancer 27: 806–807 10.1016/0277-5379(91)90197-l 1829930

[bib9] Hayward JL, Carbone PP, Heuson JC, Kumaoka S, Segaloff A, Rubens RD (1977) Assessment of response to therapy in advanced breast cancer. Cancer 39: 1289–1293 912660 10.1002/1097-0142(197703)39:3<1289::aid-cncr2820390340>3.0.co;2-f

[bib10] Howell A, Mackintosh J, Jones M, Redford J, Wagstaff J, Sellwood RA (1988) The definition of the no change category in patients treated with endocrine therapy and chemotherapy for advanced carcinoma of the breast. Eur J Cancer Clin Oncol 24(10): 1567–1672 3208800 10.1016/0277-5379(88)90046-6

[bib11] Jonat W, Kaufmann M, Blamey RW, Howell A, Collins JP, Coates A, Eiermann W, Janicke F, Njordenskold B, Forbes JF (1995) A randomised study to compare the effect of the luteinising hormone releasing hormone (LHRH) analogue goserelin with or without tamoxifen in pre- and perimenopausal patients with advanced breast cancer. Eur J Cancer 31A(2): 137–142 7718316 10.1016/0959-8049(94)00415-2

[bib12] Klijn JGM, Blamey RW, Boccardo F, Tominaga T, Duchateau L, Sylvester R (2001) Combined tamoxifen and luteinizing hormone-releasing hormone (LHRH) agonist versus LHRH agonist alone in premenopausal advanced breast cancer: a meta-analysis of four randomized trials. J Clin Oncol 19: 343–353 11208825 10.1200/JCO.2001.19.2.343

[bib13] Milla-Santos A, Milla L, Portella J, Rallo L, Pons M, Rodes E, Casanovas J, Puig-Gali M (2003) Anastrozole versus tamoxifen as first-line therapy in postmenopausal patients with hormone-dependent advanced breast cancer: a prospective, randomized, phase III study. Am J Clin Oncol 26: 317–322 12796608 10.1097/01.COC.0000047126.10522.F9

[bib14] Mouridsen H, Gershanovich M, Sun Y, Perez-Carrion R, Boni C, Monnier A, Apffelstaedt J, Smith R, Sleeboom HP, Janicke F, Pluzanska A, Dank M, Becquart D, Bapsy PP, Salminen E, Snyder R, Lassus M, Verbeek JA, Staffler B, Chaudri-Ross HA, Dugan M (2001) Superior efficacy of letrozole versus tamoxifen as first-line therapy for postmenopausal women with advanced breast cancer: results of a phase III study of the International Letrozole Breast Cancer Group. J Clin Oncol 19: 2596–2606 11352951 10.1200/JCO.2001.19.10.2596

[bib15] Nabholtz JM, Buzdar A, Pollak M, Harwin W, Burton G, Mangalik A, Steinberg M, Webster A, von Euler M for the Arimidex Study Group (2000) Anastrozole is superior to tamoxifen as first-line therapy for advanced breast cancer in postmenopausal women: results of a North American multicenter randomized trial. J Clin Oncol 18: 3758–3767 11078488 10.1200/JCO.2000.18.22.3758

[bib16] Nicholson RL, Walker KJ, Turks A, Dyas J, Plowman PN, Williams M, Blamey RW (1985) Endocrinological and clinical aspects of LH-RH action (ICI 118630) in hormone dependent breast cancer. J Steroid Biochem 23: 843–847 2934580 10.1016/s0022-4731(85)80025-x

[bib17] Robertson JF, Willsher PC, Cheung KL, Blamey RW (1997) The clinical relevance of static disease (no change) category for 6 months on endocrine therapy in patients with breast cancer. Eur J Cancer 33(11): 1774–1779 9470831 10.1016/s0959-8049(97)00178-0

[bib18] Robertson JFR, Pearson D, Price MR, Selby C, Blamey RW, Howell A (1991) Objective measurement of therapeutic response in breast cancer using tumour markers. Br J Cancer 64: 757–763 1911226 10.1038/bjc.1991.394 PMC1977677

[bib19] Robertson JFR, Walker KJ, Nicholson RI, Blamey RW (1989a) Combined endocrine effects of LHRH agonist (Zoladex) and tamoxifen (Nolvadex) therapy in premenopausal women with breast cancer. Br J Surg 76: 1262–1265 2532556 10.1002/bjs.1800761213

[bib20] Robertson JFR, Williams MR, Todd J, Nicholson RI, Morgan DA, Blamey RW (1989b) Factors predicting response of patients with advanced breast cancer to endocrine (Megace) therapy. Eur J Cancer Clin Oncol 25: 469–475 2703001 10.1016/0277-5379(89)90259-9

[bib21] Taylor CW, Green S, Dalton WS, Martino S, Rector D, Ingle JN, Robert NJ, Budd GT, Paradelo JC, Natale RB, Bearden JD, Mailliard JA, Osborne CK (1998) Multicenter randomized clinical trial of goserelin versus surgical ovariectomy in premenopausal patients with receptor-positive metastatic breast cancer: an intergroup study. J Clin Oncol 16: 994–999 9508182 10.1200/JCO.1998.16.3.994

[bib22] Williams MR, Walker KJ, Turkes A, Blamey RW, Nicholson RI (1986) The use of an LH–RH agonist (ICI 118630, Zoladex) in advanced premenopausal breast cancer. Br J Cancer 53: 629 2941044 10.1038/bjc.1986.106 PMC2001373

[bib23] Wiseman LR, Adkins JC (1998) Anastrozole. A review of its use in the management of postmenopausal women with advanced breast cancer. Drugs and Ageing 13(4): 321–332 10.2165/00002512-199813040-00008 9805213

